# Factors that influence the intent to pursue a master’s degree: evidence from Shandong Province, China

**DOI:** 10.3389/fpsyg.2024.1284277

**Published:** 2024-01-12

**Authors:** Yuhong Zhou, Yi Liu, Wenhao Xue, Xinyao Li, Zhe Yang, Zhihua Xu

**Affiliations:** ^1^School of Tourism and Geography Science, Qingdao University, Qingdao, China; ^2^School of Economics, Qingdao University, Qingdao, China; ^3^School of Economics, Ocean University of China, Qingdao, China; ^4^Institute of Marine Development, Ocean University of China, Qingdao, China

**Keywords:** intent to pursue a master’s degree, attitude, subjective norms, perceived behavioral control, risk perception, social factors

## Abstract

**Introduction:**

In recent years, the pursuit of a master’s degree has become a social phenomenon of wide concern. It is essential to understand why large number of students choose to pursue master’s degree. This study aims to empirically analyze the factors that influence the intent to pursue a master’s degree.

**Method:**

Based on the extended theory of planned behavior, this study conducts a questionnaire survey of university students in Shandong Province, which has had the highest number of people taking the postgraduate entrance examination in China for several years. A total of 440 questionnaires were finally collected, including 417 valid questionnaires. And then ordinary least squares (OLS) regression was used to analyze the factors that influence the intent to pursue a master’s degree.

**Results:**

In general, the intent to pursue a master’s degree is positively influenced by attitude (β = 0.161, *p* < 0.01) and subjective norms (β = 0.208, *p* < 0.01), and negatively influenced by risk perception (β = −0.084, *p* < 0.05). Compared with male students, female students’ intent is more likely to be influenced by risk perception (β = −0.144, *p* < 0.05) and social factors (β = 0.140, *p* < 0.05). The intent of upperclass students tends to be positively influenced by perceived behavioral control (β = 0.125, *p* < 0.05), whereas the negative impact of risk perception (β = −0.219, *p* < 0.05) on the intent is significant for underclass students. The intent of students in rural areas are more sensitive to risk perception (β = −0.194, *p* < 0.01) than those of students in cities. In private universities, social factors (β = 0.445, *p* < 0.05) significantly affect the intent to pursue a master’s degree. In ordinary public universities, the intent of students is more likely to be influenced by risk perception (β = −0.082, *p* < 0.05).

**Conclusion:**

The study is helpful to strengthen the understanding of the influencing factors of the intent to pursue a master’s degree. In general, the intent to pursue a master’s degree is mainly influenced by attitude, subjective norms and risk perception. Moreover, the influencing factors vary among different groups (e.g., female vs. male, rural areas vs. cities). Furthermore, attitude, subjective norms, perceived behavioral control, risk perception, and social factors have greater impacts on the intent of students from low-income households than those from high-income households. This study can provide policy implications for universities to take targeted educational measures to encourage students to make a choice that suits their own development after graduation.

## 1 Introduction

In recent years, the enthusiasm for pursuing a master’s degree (MD) has become the focus of social attention (Jung, 2020; Statista Research Department, 2022; Jung et al., 2023). According to China Education Online 2023 National Graduate Student Enrollment Data Survey Report, the number of applicants for the postgraduate entrance examination in 2023 was 4.74 million, which represented a new high. In fact, this number in China entered a high-growth phase since 2016, with an average annual growth rate of 14.1% from 2016 to 2023. Thus, it is essential to analyze what factors lead to such a strong intent to pursue an MD.

It is worth noting that, college students are in the exploration stage of their career (Super, 1980), and the uncertainty of identity exploration can easily lead to confusion and pressure (Shelley et al., 2020). Data from the 2022 White Paper on Learning and Development of Chinese College Students show that “confusion about the future” accounts for the largest proportion of the growing pain points of college students. As large numbers of college students are confused about their future, they may follow the trend of taking the postgraduate entrance examination, which is not based on their future development. In addition, studies have shown that many countries are “over-educated,” meaning that the level of education exceeds the job requirements (Green and Henseke, 2016). That is to say, college students who choose to pursue an MD will also face the risk of uncertainty in the job market upon graduation. Based on this, students should carefully consider whether or not to pursue an MD.

The aim of this study is to explore the factors that influence the intent to pursue an MD, and if the factors vary among different groups, so that we can put forward specific suggestions to guide undergraduates to make a choice that suits their own development after graduation.

## 2 Literature review

### 2.1 Influencing factors for pursuing an MD

The existing research shows that students’ intent to pursue an MD is affected by many factors. Some scholars believe that there is a strong correlation between intent and individual factors, such as demographic attributes, academic background during the undergraduate period, self-efficacy and career development. Jung and Lee (2019) explored the factors that influence South Korean university graduates to pursue an MD using logistic regression model and found that gender and age had an impact on students’ decisions. However, Borrego et al. (2018) investigated American engineering undergraduates, using the multiple logistic regression model, and found that the inherent background characteristics of individuals, such as gender and race, had little impact on their intent to pursue graduate study. Therefore, scholars have not reached a consensus on the impact of demographic attributes on the intent to pursue an MD. Some scholars have also studied the influence of academic background on students’ intent during undergraduate period. Most of the research results show that undergraduate academic performance, such as grade point average, has a significant positive impact on students’ decision-making (Davis et al., 2012; Liu and Lin, 2021; Zamfir et al., 2021). Some studies have found that students’ major is an important factor that affects students’ intent (Kong et al., 2015; English and Umbach, 2016). According to Cseh-Papp et al. (2023), the more satisfied students are with the quality of their education, the more likely they are to pursue a master’s degree. Self-efficacy is one of the most widely studied psychological predictors of academic perseverance (Estrada et al., 2011). Based on this, a study found that self-efficacy has a significant impact on the intent to pursue an MD. For every one-unit increase in students’ self-efficacy, students are more than 8 times more likely to plan to enroll in a master’s program relative to not attending graduate school (Borrego et al., 2018). Amani et al. (2022) used qualitative research methods to study the motivation of Tanzanian students for postgraduate study and found that the main reasons were to obtain promotions and better salaries, to make progress or changes in their careers, to develop themselves, to gain prestige or to achieve self-realization. Similar findings have been confirmed in other studies (De Los Santos et al., 2019; Jung, 2019; Makrygianni et al., 2023; Rock et al., 2023).

In addition, some external factors such as the opinions of parents and friends, family financial situation, and the prestige of the university can also influence students’ intent to pursue an MD (Buchmann and Dalton, 2002; Mullen et al., 2003; Berggren, 2006; Zhang and Tang, 2021). Several studies have found that parents’ expectations and encouragement have a positive and significant impact on students’ intent to pursue an MD (Guerin et al., 2014; To et al., 2014). Parents with higher socioeconomic status are believed more likely to provide high-quality learning environment and resources for their children than those with lower income, thus affecting students’ pursuit of master education differently (Kong et al., 2015; Posselt and Grodsky, 2017). In view of the increasing number of students who self-fund their MDs, family financial situation is an important factor in students’ decision to pursue an MD (Jung and Lee, 2019). Zamfir et al. (2021) found that university prestige or overall university ranking has a significant impact on students’ intent to pursue an MD. Specifically, students from more prestigious universities are more motivated to pursue an MD than students from less prestigious universities. As noted by English and Umbach (2016), the different types of universities that students attend during their first degree expose them to different intellectual atmospheres and resource platforms, thus affecting their intent to pursue graduate studies. Other studies have shown that mentors or teachers play an important role in students’ decisions to pursue an MD (Cruce et al., 2006; Loes and Pascarella, 2015; Hanson et al., 2016; Jung et al., 2022). On the one hand, teachers themselves have a demonstration effect; they can show students their passion for research and explain the value of an MD to students through personal experience, thus influencing students’ choices (Guerin and Ranasinghe, 2010). On the other hand, teachers can stimulate students’ interest in scientific research and encourage and support students’ pursuit of higher education, which will enhance students’ intent to pursue an MD (Guerin et al., 2014; Liu and Morgan, 2016). Davis et al. (2012) explored the factors that influence female university students to pursue graduate studies using logistic regression model and found that peer experience produces higher degree expectations, and one additional unit of peer experience increases the intent to pursue graduate studies by 3.5%. In fact, positive interactions reinforce students’ performance and aspirations, resulting in higher educational expectations for each group (Pascarella, 1985; Hubbard, 2005; To et al., 2014; Thomas et al., 2021).

### 2.2 Theoretical background

So far, previous studies have made significant contributions to the descriptive analysis of students’ decisions regarding pursuing an MD, but there is still a lack of strong theoretical analysis. Among the studies mentioned above, only a few of them involve theoretical conceptual models. The human capital theory provides a valuable framework for explaining the reasons for pursuing an MD. However, the human capital theory does not consider internal dimensions, including individual socio-economic background, personal characteristics, and academic ability (Jung and Lee, 2019). The theory of planned behavior (TPB) is one of the most commonly used and influential models for studying individual behavior(Yuriev et al., 2020). To date, the TPB has been broadly used in various fields of human life, including health (Broers et al., 2020; Rajeh, 2022), environmental protection (Arya and Chaturvedi, 2020; Sun et al., 2022), tourist behavior (Japutra et al., 2019; Ulker-Demirel and Ciftci, 2020), organizational citizenship behavior (Ma et al., 2020; Tsai et al., 2022), electronic shopping (Tang et al., 2021; Theodorou et al., 2023), and transportation (Chen, 2022; Ni et al., 2022). In terms of education, it is mainly used in innovation and entrepreneurship intention research. Attitude, subjective norms and perceived behavioral control have been found to have substantial effects on university students’ entrepreneurial intent (Shah and Soomro, 2017; Ma et al., 2020; Aliedan et al., 2022; Blanco-Mesa et al., 2023). The TPB considers both internal and external influencing factors in individual decision-making processes, making it an appropriate research foundation for this paper. However, some studies have shown that the TPB also has some limitations in explaining and predicting human behavior(Ulker-Demirel and Ciftci, 2020). Therefore, given that perceived risks and social factors may play an important role in students’ decision to pursue an MD, we use an extended model of TPB which includes risk perception and social factors to analyze the influencing factors of the intent to pursue an MD.

As mentioned above, scholars have studied the factors that influence students’ intent to pursue an MD from different perspectives and have achieved various results. However, there is still room for further study. First, few studies have analyzed the influencing factors of students’ intent to pursue an MD from the perspective of TPB. This paper introduces an extended model of TPB to systematically analyze the influence of attitude, subjective norms, perceived behavior control, risk perception and social factors on students’ intent to pursue an MD. Second, few studies have explored the differences in the factors that influence students’ intent to pursue an MD among different groups. Based on the personal characteristics of the respondents, this paper conducts a series of regression analyses to examine the differences in the influencing factors among different groups. Finally, few studies have investigated the moderating effect of family income on the influencing factors of students’ intent to pursue an MD. This paper uses the moderating effect model to analyze the moderating effect of family income on the influence of factors related to the intent to pursue an MD empirically to better understand the role of the family economic situation in students’ intent.

## 3 The analytical framework and research hypothesis

### 3.1 The TPB and the intent to pursue an MD

The TPB is a social psychological theory that focuses on the determinants of individual behavior (Ajzen, 1991). It has become one of the most prominent and popular theories for predicting and explaining human behavior (Ulker-Demirel and Ciftci, 2020; Yuriev et al., 2020). According to the theory, behavioral intent is the most important direct factor of behavior. Generally, the stronger the intent for a behavior, the more likely one is to perform the behavior (Ajzen, 1991; Xu et al., 2021). Intent depends on three key predictors: attitude toward the behavior, subjective norms, and perceived behavioral control (Ajzen, 2002). Attitude represents a summary evaluation of participating in a given behavior; Subjective norms are the social pressure perceived from significant others (e.g., family, friends, and peers) to implement specific behaviors; while the degree of ease or difficulty that an individual perceives when performing certain behaviors is represented by perceived behavioral control (Ajzen, 1991).

In terms of the intent to pursue an MD, attitude refers to students’ overall evaluation of further study. Students may perceive pursuing an MD as an investment that enhances their professional knowledge and skills through graduate study, which may lead to favorable returns (i.e., higher earnings, promotion) in the future. With regard to subjective norms, students may perceive expectations from family members, teachers, and friends that may influence their decision to pursue further education. With regard to perceived behavioral control, students evaluate the perceived ease or difficulty in obtaining knowledge and showing perseverance in the process of preparing for further education. Students who have a positive evaluation of pursuing an MD, students who perceive strong expectations from significant individuals in their lives, and students who believe they have sufficient ability to succeed in further education should have stronger intention to pursue an MD. Therefore, the following hypotheses are proposed:

Hypothesis 1: Attitude toward an MD has a positive effect on the intent to pursue an MD.

Hypothesis 2: Subjective norms have a positive effect on the intent to pursue an MD.

Hypothesis 3: Perceived behavioral control has a positive effect on the intent to pursue an MD.

### 3.2 Risk perception and the intent to pursue an MD

Risk perception refers to people’s subjective judgment of risk, which is influenced by cultural, organizational, psychological and social factors and can be quantified and predicted by a psychometric paradigm (Slovic, 1987; Vassie et al., 2005). Numerous studies have demonstrated the importance of risk perceptions for people’s behavior (Rembischevski and Caldas, 2020; Siegrist and Árvai, 2020; Yu et al., 2020). For instance, Yin and Wu (2023) found that risk perception had a significant negative impact on university students’ entrepreneurial intent. Specifically, the higher students’ perceived entrepreneurial risk, the lower their entrepreneurial motivation. Similarly, Behrisch and Gemino (2020) found that university students’ risk perception was negatively correlated with their possibility of completing overseas study experience.

In making the decision to pursue an MD, risk perception refers to the perceived risks that students may face during the process of preparing for further education, such as the risk of failing the entrance exam due to intense competition or missing out on job opportunities due to their commitment to further education. In summary, the stronger an individual’s risk perception is, the less likely the individual is to take action. Therefore, we propose the following hypothesis:

Hypothesis 4: Risk perception has a negative effect on the intent to pursue an MD.

### 3.3 Social factors and the intent to pursue an MD

Social factors cannot be ignored in regard to individual behavior choices. The expansion of higher education has become a universal phenomenon since the 20th century. The occurrence of the knowledge economy and the increment in high-skilled employment opportunities have promoted the expansion of higher education policies on a global scale (Wright and Horta, 2017; Jung et al., 2023). Brooks et al. (2021) conducted a series of 54 focus groups survey of students in six European countries, they found that many students across Europe believed that a key purpose of higher education was to prepare them for the labor market. As Jung and Li (2021) pointed out, the degree was viewed as a way to improve competitiveness in the job market. When large numbers of students attain a bachelor’s degree, the symbolic value is weakened. MD has become the “new bachelor’s degree” people nowadays compete for (Blagg, 2018). The resulting “degree devaluation” has led young people to seek higher degrees to maintain their market value, which has further fueled the “craze” to pursue an MD (Yang and Chan, 2020). In recent years, the COVID-19 pandemic has caused an unprecedented global crisis, triggering the worst recession in the world economy since the Great Depression and affecting various aspects of society (Settersten et al., 2020; Gagnon et al., 2023; Mahagamage and Marasinghe, 2023; Naseer et al., 2023). Even in the postpandemic era, global economic recovery still faces many challenges (Fan et al., 2023; Onyango, 2023). Layoffs, salary cuts, and bankruptcies occur frequently and lead directly to a decrease in employment demand in the market and a reduction in employment opportunities for university students (Yang et al., 2022; Zheng et al., 2022). In this context, postponing employment by pursuing further study is a rational choice (Peng et al., 2023; Shi, 2023). Therefore, we propose the following hypothesis:

Hypothesis 5: Social factors have a promoting effect on the intent to pursue an MD.

### 3.4 Moderating effects of family income

Numerous scholars have verified that the socioeconomic status of the family has an impact on individual career development (Fan and Williams, 2010; Aguayo et al., 2011; Flores et al., 2017; Zhang and Tang, 2021). Its influence on behavior and cognition is usually indirect rather than direct. According to trait activation theory, the effect of an individual’s traits on behavior can be regulated by his or her perception of a situation (Tett and Burnett, 2003). This theory suggests that situations can be categorized into strong situations and weak situations based on their intensity. In strong situations, individuals are subjected to clear and uniform requirements or expectations, leading to relatively consistent responses. Conversely, the requirements or expectations of individual behavior in weak situations are not clear, and the behavioral responses of individuals are mainly determined by individual differences and personality traits, resulting in greater variability. When family income is higher, individuals are in a strong situation, and even individuals with lower subjective initiative exhibit a more positive intention to pursue an MD due to the more favorable conditions provided by their parents. However, when family income is lower, the differences in individual characteristics are activated, and students’ intention to pursue further education is more significantly affected by potential factors. Therefore, we propose the following hypothesis:

Hypothesis 6: The higher family income is, the weaker the impact of potential factors on the intent to pursue an MD.

## 4 Methodology

To analyze the factors that influence the intent to pursue an MD, this paper focuses on quantitative analysis. First, the relevant data were obtained through a questionnaire survey, and then the influences of multiple factors on the intent to pursue an MD were empirically tested through multiple linear regression. Subsample analyses were conducted according to individual characteristics to explore the differences in influencing factors among groups.

### 4.1 Questionnaire design

Based on the extended model of TPB and related literature, we designed a questionnaire and invited experts to modify its expression and wording. Before the formal questionnaire was developed, a small-scale presurvey was conducted over 2 weeks, and the questionnaire was analyzed and modified according to the problems identified in the presurvey to ensure reliability. After the pilot study, the formal questionnaire survey lasted over a month. To ensure the effectiveness and timeliness of the questionnaire distribution, the questionnaire was distributed by “Questionnaire Star,” a large-scale online survey company in China.

**Figure 1:**
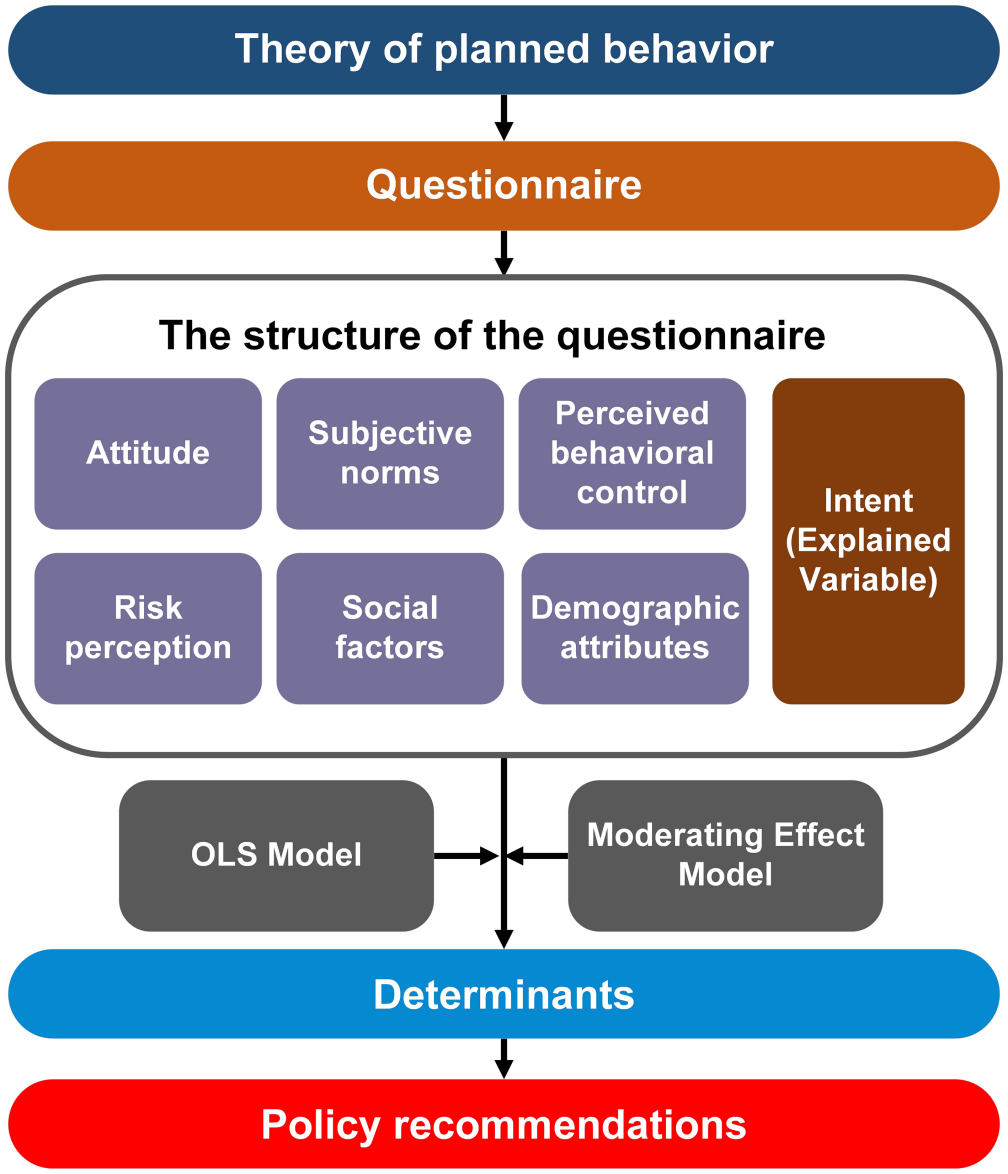
Structure of the questionnaire.

The structure of the questionnaire is presented in Figure 1. The first part examined the intent to pursue an MD, which was measured with a 5-point Likert scale ranging from 1 = low to 5 = high. The second part explored potential influencing factors for pursuing an MD. Based on the TPB, Attitude (AT), subjective norms (SN), and perceived behavioral control (PBC) were reflected by three items. Similarly, risk perception (RP) and social factors (SO) were reflected by three items. All 15 items were evaluated on a 5-point Likert scale ranging from 1 = strongly disagree to 5 = strongly agree (Supplementary Table S1). The last section was designed to identify the detailed attributes of respondents (Supplementary Table S2).

### 4.2 Sample

Shandong is a province of China with a large population of people who take the postgraduate entrance examination. In 2023, there were 441,152 students enrolled in postgraduate entrance examinations in Shandong Province, accounting for 9.3% of the total number nationally. Therefore, this paper takes students in Shandong Province as the representative survey subjects. A total of 440 samples of the questionnaires were collected through the online survey. The criteria of the sample include: for undergraduates of different grades in universities in Shandong Province; the types of universities include 985/211 universities, ordinary public universities and private universities. If the questionnaire does not meet the above requirements or has not been completed, it will be invalid. Finally, 417 samples were identified as valid samples.

The final samples included 188 male students and 229 female students. Among them, 112 were senior students, 179 were juniors, 91 were sophomores and 35 were freshmen. The percentage of upperclass students (juniors and seniors) was higher than the percentage of freshmen and sophomores. Regarding the types of universities, there were 86 students from 985 and 211 universities, 302 students from ordinary public universities, and 29 students from private universities. The distribution of the questionnaire was basically in line with the scale of various types of universities in Shandong Province. In terms of majors, there were 67 students in literature and history (L&H), 93 in economics and management (E&M), 171 in science and engineering (S&E), 24 in arts and physical education (A&P), 48 in medicine (Med), and 14 in other majors (Oth).

Before the analysis, the questionnaire used Cronbach’s α to test the reliability of the answers to quantitative data. The test results showed that the Cronbach’s α for all five potential influencing factors were greater than 0.7 (Supplementary Table S1), and the overall Cronbach’s α was 0.841, indicating that the reliability of the research data is good.

### 4.3 Estimation model

Ordinary least squares (OLS) regression was used to analyze the impacts of attitude, subjective norms, perceived behavioral control, risk perception, and social factors on the intent to pursue an MD. The model (Equation 1) was set as follows:

(1) $$\begin{array}{l} Y_{i}=\beta_{0}+\beta_{1}AT_{i}+\beta_{2}SN_{i}+\beta_{3}\mathrm{PB}C_{i}+\beta_{4}RP_{i}+\\ \beta_{5}SO_{i}+\beta_{6}X_{i}+\varepsilon_{i} \end{array}$$

where *Y_i_* is respondent *I*’s intent to pursue an MD, *AT_i_* is respondent *I*’s attitude toward pursuing an MD, *SN_i_* is respondent *I*’s subjective norms, *PBC_i_* is respondent *I*’s perceived behavioral control, *RP_i_* is respondent *I*’s risk perception, and *SO_i_* is the social factors faced by respondent *i*. *X_i_* is a set of control variables, which are respondent *I*’s personal characteristics. ε _i_ denotes a random error term. The scores of five potential factors (AT, SN, PBC, RP, SO) were obtained by confirmatory factor analysis.

## 5 Results

### 5.1 Descriptive statistics

**Figure 2:**
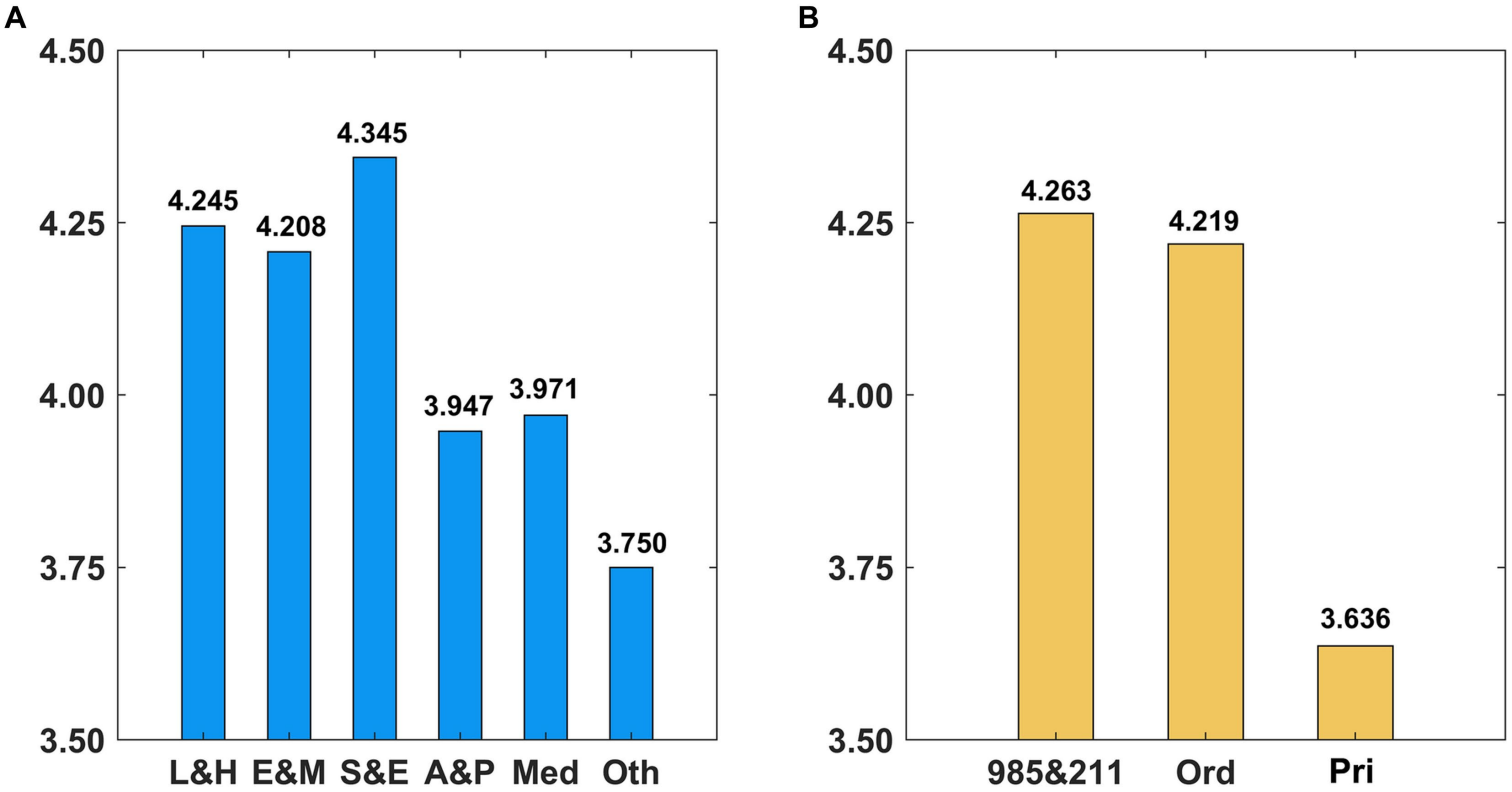
The intent to pursue an MD among different groups. **(A)** Major category. **(B)** University type.

From descriptive statistics in Supplementary Table S3, it can be found that the overall intent of the respondents to pursue an MD was strong, with an average score of 4.237, which is consistent with the fact that Shandong Province currently contains the largest population taking the postgraduate entrance examination among all provinces in China. To further observe the heterogeneity of the respondents’ intent to pursue an MD, we grouped the sample by major and university type. As presented in Figure 2A, science and engineering students had the highest average score of 4.345, followed by literature and history, economic management, medicine, arts and sports, and others. According to the types of universities of undergraduates, as presented in Figure 2B, students from 985 and 211 universities had the strongest intent to pursue an MD, followed by students from ordinary public universities. Students from private universities had the weakest intent to pursue an MD.

### 5.2 Estimation of basic regression

Table 1 shows the impact of influencing factors on the intent to pursue an MD. To ensure the robustness of the regression results, potential influencing factors were gradually added to the model. The results showed that attitude and subjective norms had significantly positive impacts on the intent to pursue an MD, with coefficients of 0.161 (*p* < 0.01) and 0.208 (*p* < 0.01), respectively, which verifies Hypothesis 1 and Hypothesis 2. While risk perception had a significantly negative impact on the intent to pursue an MD, with a coefficient of −0.084 (*p* < 0.05), which verifies Hypothesis 4. In general, perceived behavioral control and social factors had no significant impact on the intent to pursue an MD.

**Table 1:** The results of the benchmark regression.

	(1)	(2)	(3)	(4)	(5)
AT	0.258***	0.181***	0.171***	0.171***	0.161***
	(6.468)	(4.692)	(4.199)	(4.207)	(4.105)
SN		0.236***	0.221***	0.212***	0.208***
		(5.924)	(5.399)	(5.155)	(5.060)
PBC			0.050	0.043	0.037
			(1.080)	(0.938)	(0.810)
RP				−0.063*	−0.084**
				(−1.861)	(−2.167)
SO					0.067
					(1.616)
Gender	0.100	0.060	0.051	0.042	0.045
	(1.379)	(0.854)	(0.717)	(0.584)	(0.628)
Graduation	−0.005	−0.010	−0.007	−0.004	−0.003
	(−0.113)	(−0.210)	(−0.138)	(−0.093)	(−0.073)
Honor	−0.011	−0.050	−0.048	−0.047	−0.034
	(−0.083)	(−0.415)	(−0.397)	(−0.389)	(−0.293)
Performance	0.227***	0.184***	0.169***	0.162**	0.158**
	(3.368)	(3.063)	(2.704)	(2.582)	(2.552)
Internship	−0.084	−0.101	−0.104	−0.095	−0.099
	(−1.035)	(−1.311)	(−1.355)	(−1.248)	(−1.298)
Income	0.077	0.066	0.059	0.050	0.034
	(1.634)	(1.463)	(1.299)	(1.077)	(0.711)
City	0.005	−0.022	−0.023	−0.021	−0.010
	(0.058)	(−0.278)	(−0.286)	(−0.266)	(−0.130)
Constant	13.938	23.357	16.907	12.619	10.613
	(0.146)	(0.246)	(0.174)	(0.129)	(0.111)
R-squared	0.181	0.252	0.255	0.260	0.266
Observations	417	417	417	417	417

AT, Attitude; SN, Subjective norms; PBC, Perceived behavioral control; RP, Risk perception; SO, Social factors. Robust t-statistics in parentheses. ***p < 0.01, **p < 0.05, *p < 0.1.

Among the control variables, students’ academic performance positively impact their intent to pursue an MD at a significance level of 5%. This is consistent with expectations for human capital investment decisions, since theoretically higher academic performance increases the demand for graduate education (English and Umbach, 2016). Students who achieve better grades in college have a greater expectation of improving their research ability and practical employment ability through graduate study.

### 5.3 Robustness check

In the benchmark regression, five potential influencing factors were obtained by confirmatory factor analysis. To check the robustness of the regression results, this section assigns a value to the five potential influencing factors by averaging the scores of the subitems. The regression results are showed in Table 2. They are basically consistent with the baseline regression results after replacing the measurement method of influencing factors, which confirms the robustness of the research results.

**Table 2:** Robustness check.

	(1)	(2)	(3)	(4)	(5)
AT	0.478***	0.332***	0.311***	0.312***	0.292***
	(6.064)	(4.444)	(3.944)	(3.967)	(3.852)
SN		0.436***	0.407***	0.391***	0.384***
		(6.043)	(5.502)	(5.257)	(5.165)
PBC			0.089	0.075	0.063
			(1.084)	(0.931)	(0.783)
RP				−0.063*	−0.083**
				(−1.903)	(−2.197)
SO					0.078
					(1.601)
Gender	0.099	0.058	0.049	0.040	0.043
	(1.358)	(0.831)	(0.690)	(0.557)	(0.600)
Graduation	−0.003	−0.009	−0.005	−0.003	−0.002
	(−0.054)	(−0.182)	(−0.105)	(−0.062)	(−0.042)
Honor	−0.012	−0.052	−0.049	−0.048	−0.037
	(−0.093)	(−0.431)	(−0.407)	(−0.404)	(−0.310)
Performance	0.228***	0.184***	0.169***	0.161**	0.158**
	(3.364)	(3.059)	(2.682)	(2.563)	(2.531)
Internship	−0.086	−0.104	−0.107	−0.097	−0.100
	(−1.065)	(−1.346)	(−1.388)	(−1.277)	(−1.308)
Income	0.078*	0.066	0.059	0.050	0.034
	(1.660)	(1.462)	(1.293)	(1.068)	(0.696)
City	0.003	−0.025	−0.025	−0.024	−0.013
	(0.033)	(−0.307)	(−0.314)	(−0.294)	(−0.164)
Constant	6.206	17.379	10.282	6.438	4.429
	(0.065)	(0.183)	(0.106)	(0.066)	(0.046)
R-squared	0.172	0.246	0.249	0.255	0.261
Observations	417	417	417	417	417

AT, Attitude; SN, Subjective norms; PBC, Perceived behavioral control; RP, Risk perception; SO, Social factors. Robust t-statistics in parentheses. ***p < 0.01, **p < 0.05, *p < 0.1.

### 5.4 Heterogeneous impacts

The influencing factors of the intent to pursue an MD may vary among different groups. Therefore, in this section, four aspects of heterogeneity are analyzed.

#### 5.4.1 Gender

Students of different genders may take different factors into consideration when choosing whether to pursue an MD. We conducted subsample analysis by gender with the estimation model in Section 4.3. Results are presented in Table 3. The main factors that affect female students’ intent to pursue an MD include attitude ( $\beta_{1}$ = 0.155, *p* < 0.05), subjective norms ( $\beta_{2}$ = 0.193, *p* < 0.01), risk perception ( $\beta_{4}$ = −0.144, *p* < 0.05) and social factors ( $\beta_{5}$ = 0.140, *p* < 0.05); that is, both endogenous and exogenous factors have a significant impact on their intent to pursue an MD. While male students’ intent is just significantly affected by attitude and subjective norms, with coefficients of 0.148 (*p* < 0.01) and 0.232 (*p* < 0.01).

**Table 3:** The impact of different factors on the intent to pursue an MD by gender.

	(1)	(2)
	Female	Male
AT	0.155**	0.148***
	(2.38)	(3.34)
SN	0.193***	0.232***
	(3.64)	(3.78)
PBC	0.022	0.056
	(0.40)	(0.73)
RP	−0.144**	−0.010
	(−2.58)	(−0.18)
SO	0.140**	−0.016
	(2.20)	(−0.31)
Control variables	Yes	Yes
R-squared	0.230	0.330
Observations	229	188

AT, Attitude; SN, Subjective norms; PBC, Perceived behavioral control; RP, Risk perception; SO, Social factors. Robust t-statistics in parentheses. ***p < 0.01, **p < 0.05, *p < 0.1.

#### 5.4.2 Year of graduation

Due to the different learning experiences of students in different grades, the influencing factors of the intent to pursue an MD may also be different. According to the respondents’ graduation time, we divided the sample into upperclass students (junior and seniors) and underclass students (freshman and sophomore). The results are presented in Table 4. For students with different graduation years, heterogeneity is mainly manifested in perceived behavioral control and risk perception. The perceived behavioral control of upperclass students positively impacts their intent to pursue an MD, with a coefficient of 0.125 (*p* < 0.05), whereas the impact of the perceived behavioral control of underclass students on their intent to pursue an MD is negative and not significant. Additionally, the negative impact of risk perception on the intent to pursue an MD is more significant among underclass students, with a coefficient of −0.219 (*p* < 0.05).

**Table 4:** The impact of different factors on the intent to pursue an MD by graduation year.

	(1)	(2)
	Upperclassman	Underclassman
AT	0.121**	0.255***
	(2.57)	(2.92)
SN	0.210***	0.170**
	(4.40)	(1.98)
PBC	0.125**	−0.096
	(2.33)	(−1.07)
RP	−0.033	−0.219**
	(−0.79)	(−2.32)
SO	0.046	0.128
	(1.03)	(1.48)
Control variables	Yes	Yes
R-squared	0.322	0.264
Observations	291	126

AT, Attitude; SN, Subjective norms; PBC, Perceived behavioral control; RP, Risk perception; SO, Social factors. Robust t-statistics in parentheses. ***p < 0.01, **p < 0.05, *p < 0.1.

#### 5.4.3 Home address

Due to the different living environment of students from rural areas and cities, there may be differences in the influencing factors of the intent to pursue an MD. According to home address, we divided the sample into two groups, rural areas and cities. The results are presented in Table 5. The differences in the influencing factors in rural areas and cities mainly involve risk perception. The intent of rural students is more sensitive to risks, with a coefficient of −0.194 (*p* < 0.01).

**Table 5:** The impact of different factors on the intent to pursue an MD by home address.

	(1)	(2)
	Rural area	City
AT	0.146*	0.168***
	(1.76)	(3.48)
SN	0.224***	0.192***
	(3.25)	(3.88)
PBC	0.058	0.021
	(0.78)	(0.36)
RP	−0.194***	−0.047
	(−2.66)	(−1.05)
SO	0.081	0.070
	(1.42)	(1.38)
Control variables	Yes	Yes
R-squared	0.319	0.251
Observations	136	281

AT, Attitude; SN, Subjective norms; PBC, Perceived behavioral control; RP, Risk perception; SO, Social factors. Robust t-statistics in parentheses. ***p < 0.01, **p < 0.05, *p < 0.1.

#### 5.4.4 Type of undergraduate university

Due to the differences in the learning environment of students in different types of undergraduate universities, the influencing factors of the intent to pursue an MD may be different. We divided the sample according to the types of undergraduate universities that the respondents attended, and the regression results are presented in Table 6. The discrepancy of impacts is mainly reflected in attitude, risk perception and social factors. The attitude of students in private universities has no significant impact on their intent to pursue an MD. Students’ intent to pursue an MD in private universities is significantly positively influenced by social factors, with a coefficient of 0.445 (*p* < 0.05). While the students’ intent to pursue an MD in ordinary public universities tends to be vulnerable to risk perception, with a coefficient of −0.082 (*p* < 0.05).

**Table 6:** The impact of different factors on the intent to pursue an MD by the type of undergraduate university.

	(1)	(2)	(3)
	“985&211” university	Ordinary public university	Private university
AT	0.179**	0.186***	0.204
	(2.43)	(4.10)	(0.85)
SN	0.254**	0.137***	0.605***
	(2.53)	(3.44)	(3.49)
PBC	−0.047	0.068	−0.089
	(−0.46)	(1.25)	(−0.37)
RP	−0.026	−0.082**	−0.414
	(−0.32)	(−1.99)	(−1.45)
SO	−0.025	0.048	0.445**
	(−0.30)	(1.10)	(2.46)
Control variables	Yes	Yes	Yes
R-squared	0.381	0.245	0.732
Observations	86	302	29

AT, Attitude; SN, Subjective norms; PBC, Perceived behavioral control; RP, Risk perception; SO, Social factors. Robust t-statistics in parentheses. ***p < 0.01, **p < 0.05, *p < 0.1.

### 5.5 Moderating effect

**Figure 3:**
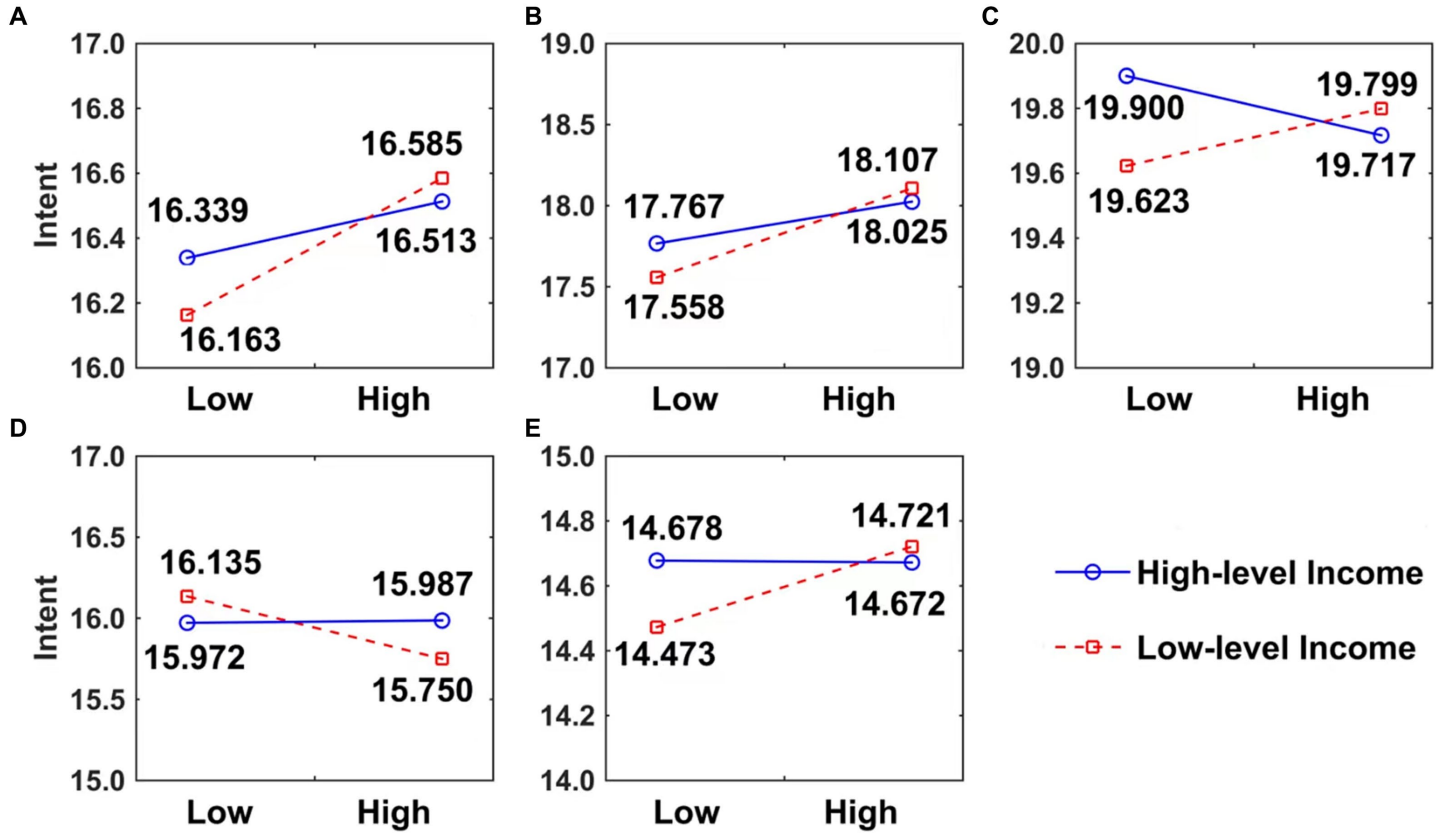
Moderating effect of family income on the impacts of influencing factors on the intent to pursue an MD. **(A)** AT. **(B)** SN. **(C)** PBC. **(D)** RP. **(E)** SO.

According to the theoretical analysis in Section 3.4, family income may have a negative moderating effect on the intent to pursue an MD. Referring to Xue et al. (2023), the interaction terms of family income and potential factors were added to the model in Section 4.3, and the results are presented in Table 7. Family income negatively moderates the impacts of all five potential factors on the intent to pursue an MD. This means that the impacts of these influencing factors on the intent to pursue an MD are greater in the group with low family income than in the group with high family income, which verifies Hypothesis 6. In addition, this section presents a slope diagram of the moderating effect. As shown in Figure 3, the influencing factors have steeper slopes in low-income families, which further indicates that family income negatively moderates the impact of potential factors on the intent to pursue an MD.

**Table 7:** Moderating effect of family income on the impact of influencing factors on the intent to pursue an MD.

	(1)	(2)	(3)	(4)	(5)
AT	0.325***	0.160***	0.167***	0.159***	0.159***
	(3.293)	(4.039)	(4.206)	(4.042)	(4.098)
SN	0.208***	0.410***	0.206***	0.203***	0.214***
	(5.044)	(4.564)	(4.953)	(4.935)	(5.202)
PBC	0.037	0.030	0.255***	0.038	0.031
	(0.801)	(0.648)	(2.605)	(0.839)	(0.678)
RP	−0.085**	−0.088**	−0.092**	−0.377***	−0.087**
	(−2.192)	(−2.276)	(−2.365)	(−3.401)	(−2.254)
SO	0.067	0.074*	0.068*	0.080*	0.241**
	(1.645)	(1.785)	(1.657)	(1.914)	(2.203)
AT × income	−0.076**				
	(−1.969)				
SN × income		−0.090**			
		(−2.570)			
PBC × income			−0.111**		
			(−2.535)		
RP × income				0.123***	
				(3.066)	
SO × income					−0.078*
					(−1.719)
Control variables	Yes	Yes	Yes	Yes	Yes
R-squared	0.271	0.274	0.276	0.279	0.271
Observations	417	417	417	417	417

AT, Attitude; SN, Subjective norms; PBC, Perceived behavioral control; RP, Risk perception; SO, Social factors. Robust t-statistics in parentheses. ***p < 0.01, **p < 0.05, *p < 0.1.

## 6 Discussion

This study aimed to examine the factors that influence the intent to pursue an MD, using a questionnaire survey of university students in Shandong Province. The results revealed that in general, the intent to pursue an MD is significantly influenced by attitude, subjective norms and risk perception. But the influencing factors vary among different groups. In addition, family income negatively moderates the impact of potential factors on the intent to pursue an MD.

Students’ attitude was positively correlated with their intent to pursue an MD, which indicates that the deeper students’ understanding is of the utility that can be brought by graduate study, the stronger their intent to pursue an MD. This result is consistent with what Makrygianni et al. (2023) showed in a study among Greek pharmacy students. Students may consider the cost and time incurred in graduate studies as an investment, and expect that the knowledge and skills acquired in such studies will bring favorable returns in the future (Liu, 2010), such as getting what students perceive as better jobs (Hovdhaugen and Ulriksen, 2023). This view is consistent with human capital theory (Checchi, 2006).

There is a significantly positive correlation between students’ subjective norms and their intent to pursue an MD. Parents have a significant impact on structuring the educational opportunities of students (Jacobs and Harvey, 2006). In the process of students’ growth, parents tend to guide or intervene in students’ choices of further education and even majors. Most students rely on material and spiritual support from their parents (To et al., 2014). Similarly, parents’ wishes also play an important role when students have to decide whether to pursue an MD. This result is consistent with previous research showing that parental expectations are an important influencing factor in students’ choices to pursue higher degree (Guerin et al., 2014). In addition, encouragement from friends is also an important motivation for pursuing an MD, which is in line with previous studies (Davis et al., 2012; To et al., 2014). However, the findings in this study contradicted those reported by Hovdhaugen and Ulriksen (2023). Based on a small-scale online survey, Hovdhaugen and Ulriksen (2023) conducted a study on the reasons why students in Norway and Denmark pursue an MD. They suggested that family and friends’ expectations were perceived as the least important factor. The difference can be attributed to different cultural backgrounds, particularly the influence of Confucianism originating from Shandong.

The risk perception of students and their intent to pursue an MD are negatively correlated. This is consistent with the findings of Borrego et al. (2018). The opportunity cost of time and fierce competition for pursuing an MD may make it difficult for students to choose graduate studies. The higher the perceived risk of this loss, the greater the possibility that students will give up graduate study and choose employment or start their own businesses when they weigh the benefits and harms. Moreover, we found that the impact of risk perception on female students is more significant than the influence on male students. Despite the progress of social concepts and the improvement of laws and regulations prohibiting gender discrimination, there are still traditional concepts, educational prejudices and tendencies of the mass media to have low expectations and enthusiasm for women. Women may be more inclined to pay attention to the risk factors for failure on the postgraduate entrance examination than men. Additionally, the intent of rural students is more sensitive to risks. Due to the poor material conditions of rural students, the income loss caused by graduate study rather than employment may have a more serious impact on their families.

The perceived behavioral control of students has not significant impact on their intent to pursue an MD in general. As students’ subjective evaluation of their own resources, knowledge, abilities, and preparation for postgraduate entrance examination, perceived behavioral control reflects students’ belief in their ability to face current challenges. Generally, students who believe that they are prepared or can be prepared are more proactive and confident when faced with challenges and have a stronger intent to pursue an MD (Borrego et al., 2018). This is similar to the findings of Steinmayr et al. (2019). However, students’ sense of self-efficacy may change during the lengthy process of preparation for the postgraduate entrance examination. We found that the perceived behavioral control of upperclass students positively impacts their intent to pursue an MD, whereas the impact of the perceived behavioral control of underclass students on their intent to pursue an MD is negative and not significant. This may be because senior students are closer to graduation, and their subjective beliefs about their own abilities and preparation are more closely related to reality.

The impact of social factors on students’ intent to pursue an MD is not significant on the whole. However, some previous studies have indicated that social factors have a significant impact on the willingness to pursue an MD (Yang and Chan, 2020; Zamfir et al., 2021). With the development of education, employment pressure and degree inflation have become increasingly obvious, and graduate study is regarded as a way to delay employment pressure. It can improve students’ knowledge so that they have greater advantages in future competition for employment. However, the same social situation can be interpreted differently by different students. Their responses to the social environment can be complex and varied. We found that the social environment has a more obvious impact on female students. The reason may be that women are faced with more gender discrimination in the process of job hunting (Albert, 2000). Furthermore, students’ intent to pursue an MD in private universities is positively influenced by social factors, which may be related to the school discrimination they face while job hunting. They are under greater employment pressure and thus are more sensitive to changes in the social environment.

Family income have a significant moderating effect on the intent to pursue an MD. Some scholars have pointed out that family income has a direct influence on the willingness to pursue an MD. Most would agree that students from low-income families have significantly lower willingness to pursue an MD compared to students from high-income families (Jacobs and Harvey, 2006; Kong et al., 2015). However, there is limited literature that analyzes the moderating effect of family income on the influence of willingness to pursue an MD. This study found that, family income negatively moderates the impact of potential factors on the intent to pursue an MD. A possible reason is that in high-income families in which parents provide more convenient conditions, students may choose to pursue an MD without carefully weighing all factors. In contrast, for low-income families, students need to carefully weigh various factors according to their own situation before making choices, so their intent to pursue an MD may be different. This is also reflected in the statistics of the intent of students with different family to pursue an MD. Regarding the average value of intent, the value of the high-income family group (4.411) is higher than that of the low-income family group (4.139), while the standard deviation of the intent to pursue an MD is lower in the high-income family group (0.635) than in the low-income family group (0.868).

### 6.1 Research limitations and future prospects

This study tries to explore the factors that influence the intent to pursue an MD, and it has some limitations.

First, although this study investigated the intent of students of different grades to pursue an MD, tracking survey was not conducted due to data limitations. For individual students, the intent to pursue an MD may fluctuate in different periods, and the influencing factors may also be different. It will be an interesting study to analyze the intent of individual students in different periods of university through tracking survey.

Second, due to the limitation of time and energy, this study only sampled the intent and potential influencing factors of college students in Shandong province of China, and obtained 417 valid questionnaires. It has not been studied whether there are different factors affecting the intent to pursue an MD in other regions of China. The sample scope can be expanded in future study to further improve the rationality and representativeness of samples, and make the research results more abundant and convincing.

### 6.2 Implications

The research results in this paper show that subjective norms have a significant positive impact on students’ intent to pursue an MD. This suggests that the intent of students to pursue an MD can be easily influenced by external pressure, especially from parents. In this regard, students should learn to think independently as early as possible, recognize their actual needs, cultivate subjective initiative, and not blindly follow trends or choose further study only to delay employment. Furthermore, universities should strengthen guidance for students’ career planning, help students make clear career plans according to their personality and specialties, and encourage students to develop in diversified ways.

The results of this paper show that risk perception has a significantly negative impact on the intent to pursue an MD. Fear of failure and missing job opportunities may reduce students’ willingness to pursue an MD, and the long preparation period for the postgraduate entrance examination can produce psychological pressure on students. Therefore, universities should provide psychological support services to reduce students’ anxiety and enhance their confidence. At the same time, establishing a peer support network for students can provide encouragement and guidance throughout the preparation process. In addition, recruiters should increase the scale of spring recruitment in the context of the popularity of pursuing MDs to relieve the pressure on undergraduates who are worried about missing good job opportunities.

Female students’ intent to pursue an MD is influenced by risk perception and social factors. Possible reasons for this are that women are susceptible to gender discrimination in the job-seeking process, which makes female students’ perception of the risks of pursuing an MD stronger and more sensitive to the influences of the social environment. Therefore, it is necessary to actively publicize and implement laws and regulations to protect women’s employment rights and interests, establish and improve public service mechanisms to promote women’s employment, improve information matching in the labor market, and establish a public service center for employment according to the characteristics of women’s employment in certain regions. Moreover, universities should pay more attention to female students’ psychological counseling, help students build confidence, and provide constructive suggestions for students to choose between graduate study and job applications.

The results also show that the risk perception of students in rural areas has a greater influence on their intent to pursue an MD than it does for students in cities. Because students in rural areas have poorer material conditions, families may prefer that students work as early as possible to support the family, which gives students in rural areas a stronger risk perception of further study. In this regard, the government should increase its support and investment in rural areas, raise the income level of farmers, and strengthen social security and public services so that students in rural areas can reduce their concerns. Additionally, universities should increase the support of scholarships for students from rural areas.

With increased in family income, the influences of attitude, subjective norms, perceived behavioral control, risk perception and social factors on the intent to pursue an MD are weakened. This indicates that students with high family income may not carefully weigh the advantages and disadvantages of pursuing an MD according to their actual situations. Therefore, universities should improve training in undergraduate education, actively support students’ practice and training, and assist students in integrating into society. Parents should encourage students to make educational investment choices that suit the students’ needs instead of aimlessly choosing postgraduate entrance examinations.

## 7 Conclusion

Based on the extended model of TPB, this paper systematically analyzed the influencing factors of the intent to pursue an MD by conducting a questionnaire survey in universities in Shandong Province. The results show that, (1) on the whole, the intent to pursue an MD is mainly influenced by attitude, subjective norms and risk perception. (2) There are differences among groups. In contrast to male students, female students’ intent to pursue an MD tends to be influenced by risk perception and social factors. Upperclass students’ intent is influenced by perceived behavioral control, while underclass students’ intent is negatively affected by risk perception. Students in rural areas are more sensitive to risk perception than students in cities. The intent of students in private universities is influenced by social factors, while the intent of ordinary public university students is more influenced by risk perception. (3) Family income negatively moderates the impact of potential factors on the intent to pursue an MD. Finally, the study provides indications for practice on guiding undergraduates to make a choice that suits their own development after graduation.

Future research may focus on some key areas. First, it will be helpful to analyze the intent of individual students to pursue an MD in different periods of university through tracking survey. Second, the sample scope should be expanded to more regions to further improve the rationality and representativeness of samples, and make the research results rich and convincing.

## Data availability statement

The original contributions presented in the study are included in the article/Supplementary material, further inquiries can be directed to the corresponding authors.

## Ethics statement

The studies involving humans were approved by Qingdao University. The studies were conducted in accordance with the local legislation and institutional requirements. The participants provided their written informed consent to participate in this study.

## Author contributions

YZ: Conceptualization, Data curation, Writing – original draft. YL: Formal analysis, Methodology, Writing – original draft. WX: Formal analysis, Visualization, Writing – original draft. XL: Investigation, Methodology, Writing – original draft. ZY: Data curation, Writing – original draft, Writing – review & editing. ZX: Conceptualization, Supervision, Writing – review & editing.

## References

[ref1] Aguayo D. Herman K. Ojeda L. Flores L. Y. (2011). Culture predicts Mexican Americans’ college self-efficacy and college performance. J. Divers. High. Educ. 4, 79–89. doi: 10.1037/a0022504

[ref2] Ajzen I. (1991). The theory of planned behavior. Organ. Behav. Hum. Decis. Process. 50, 179–211. doi: 10.1016/0749-5978(91)90020-t

[ref3] Ajzen I. (2002). Perceived behavioral control, self-efficacy, locus of control, and the theory of planned behavior. J. Appl. Soc. Psychol. 32, 665–683. doi: 10.1111/j.1559-1816.2002.tb00236.x

[ref4] Albert C. (2000). Higher education demand in Spain: the influence of labour market signals and family background. High. Educ. 40, 147–162. doi: 10.1023/a:1004070925581

[ref5] Aliedan M. M. Elshaer I. A. Alyahya M. A. Sobaih A. E. E. (2022). Influences of university education support on entrepreneurship orientation and entrepreneurship intention: application of theory of planned behavior. Sustainability (Basel) 14:13097. doi: 10.3390/su142013097

[ref6] Amani J. Myeya H. Mhewa M. (2022). Understanding the motives for pursuing postgraduate studies and causes of late completion: supervisors and supervisees’ experiences. SAGE Open 12:215824402211095. doi: 10.1177/21582440221109586

[ref7] Arya B. Chaturvedi S. (2020). Extending the theory of planned behaviour to explain energy saving behaviour. Environ. Clim. Technol. 24, 516–528. doi: 10.2478/rtuect-2020-0032

[ref8] Behrisch T. Gemino A. (2020). Sensation seekers who learn abroad: exploring the role of risk perception in co-op students’ international plans. Asia-Pacific J. Cooper. Educ. 21, 117–129.

[ref9] Berggren C. (2006). Labour market influence on recruitment to higher education-gender and class perspectives. High. Educ. 52, 121–148. doi: 10.1007/s10734-004-5793-y

[ref10] Blagg K. (2018). The rise of master’s degrees: master’s programs are increasingly diverse and online. Washington, DC: Urban Institute.

[ref11] Blanco-Mesa F. Niño-Amézquita D. Gutiérrez-Ayala J. (2023). Entrepreneurial intention among Colombian university students: a theory of planned behavior analysis in Colombia. Cuad. Gest. doi: 10.5295/cdg.221858fb

[ref12] Borrego M. Knight D. B. Gibbs K. Crede E. (2018). Pursuing graduate study: factors underlying undergraduate engineering students’ decisions. J. Eng. Educ. 107, 140–163. doi: 10.1002/jee.20185

[ref13] Broers V. J. V. Van den Broucke S. Luminet O. (2020). Determinants of prebiotic vegetable consumption: the extended theory of planned behaviour. Arch. Public Health 78:27. doi: 10.1186/s13690-020-00408-z, PMID: 32435478 PMC7218641

[ref14] Brooks R. Gupta A. Jayadeva S. Abrahams J. (2021). Students’ views about the purpose of higher education: a comparative analysis of six European countries. High. Educ. Res. Dev. 40, 1375–1388. doi: 10.1080/07294360.2020.1830039

[ref15] Buchmann C. Dalton B. (2002). Interpersonal influences and educational aspirations in 12 countries: the importance of institutional context. Sociol. Educ. 75, 99–122. doi: 10.2307/3090287

[ref16] Checchi D. (2006). The economics of education. Cambridge: Cambridge University Press.

[ref17] Chen X. (2022). Predicting college students’ bike-sharing intentions based on the theory of planned behavior. Front. Psychol. 13:836983. doi: 10.3389/fpsyg.2022.836983, PMID: 35310235 PMC8928168

[ref18] Cruce T. M. Wolniak G. C. Seifert T. A. Pascarella E. T. (2006). Impacts of good practices on cognitive development, learning orientations, and graduate degree plans during the first year of college. J. Coll. Stud. Dev. 47, 365–383. doi: 10.1353/csd.2006.0042

[ref19] Cseh-Papp I. Varga E. Juhász T. (2023). Examining the attitudes towards further education of students in the bachelor training programmes of higher education. Int. J. Educ. Manag. 37, 1125–1141. doi: 10.1108/IJEM-07-2022-0246

[ref20] Davis S. D. Amelink C. Hirt J. B. Miyazaki Y. (2012). Women’s educational opportunities: factors that influence their graduate school aspirations. J. Women High. Educ. 5, 141–165. doi: 10.1515/njawhe-2012-1111

[ref21] De Los Santos P. J. Gazo P. F. Ordóñez J. L. (2019). Analysis of the motivations and expectations of master’s students in education. Educar 55, 325–341. doi: 10.5565/rev/educar.1016

[ref22] English D. Umbach P. D. (2016). Graduate school choice: an examination of individual and institutional effects. Rev. High. Educ. 39, 173–211. doi: 10.1353/rhe.2016.0001

[ref23] Estrada M. Woodcock A. Hernandez P. R. Schultz P. W. (2011). Toward a model of social influence that explains minority student integration into the scientific community. J. Educ. Psychol. 103, 206–222. doi: 10.1037/a0020743, PMID: 21552374 PMC3087606

[ref24] Fan W. Anser M. K. Nasir M. H. Nazar R. (2023). Uncertainty in firm innovation scheme and impact of green fiscal policy; economic recovery of Chinese firms in the post-Covid-19 era. Econ. Anal. Policy 78, 1424–1439. doi: 10.1016/j.eap.2023.04.002

[ref25] Fan W. Williams C. M. (2010). The effects of parental involvement on students’ academic self-efficacy, engagement and intrinsic motivation. Educ. Psychol. 30, 53–74. doi: 10.1080/01443410903353302

[ref26] Flores L. Y. Navarro R. L. Ali S. R. (2017). The state of SCCT research in relation to social class. J. Career Assess. 25, 6–23. doi: 10.1177/1069072716658649

[ref27] Gagnon J. E. Kamin S. B. Kearns J. (2023). The impact of the COVID-19 pandemic on global GDP growth. J. Jpn. Inst. Econ. 68:101258. doi: 10.1016/j.jjie.2023.101258, PMID: 37012983 PMC10030258

[ref28] Green F. Henseke G. (2016). Should governments of OECD countries worry about graduate underemployment? Oxford Rev. Econ. Policy 32, 514–537. doi: 10.1093/oxrep/grw024

[ref29] Guerin C. Jayatilaka A. Ranasinghe D. (2014). Why start a higher degree by research? An exploratory factor analysis of motivations to undertake doctoral studies. High. Educ. Res. Dev. 34, 89–104. doi: 10.1080/07294360.2014.934663

[ref30] Guerin C. Ranasinghe D. (2010). Why I wanted more: inspirational experiences of the teaching-research nexus for engineering undergraduates. J. Univ. Teach. Learn. Pract. 7, 117–139. doi: 10.53761/1.7.2.8

[ref31] Hanson J. M. Paulsen M. B. Pascarella E. T. (2016). Understanding graduate school aspirations: the effect of good teaching practices. High. Educ. 71, 735–752. doi: 10.1007/s10734-015-9934-2

[ref32] Hovdhaugen E. Ulriksen L. (2023). The historic importance of degree structure: a comparison of bachelor to master transitions in Norway and Denmark. Eur. Educ. Res. J. 22, 198–215. doi: 10.1177/14749041211041230

[ref33] Hubbard L. (2005). The role of gender in academic achievement. Int. J. Qual. Stud. Educ. 18, 605–623. doi: 10.1080/09518390500224887

[ref34] Jacobs N. Harvey D. (2006). Do parents make a difference to children’s academic achievement? Differences between parents of higher and lower achieving students. Educ. Stud. 31, 431–448. doi: 10.1080/03055690500415746

[ref35] Japutra A. Loureiro S. M. C. Molinillo S. Ekinci Y. (2019). Traveller’s mindsets and theory of planned behaviour. Tour. Manag. Perspect. 30, 193–196. doi: 10.1016/j.tmp.2019.02.011

[ref36] Jung J. (2019). Learning experience and academic identity building by master’s students in Hong Kong. Stud. High. Educ. 46, 782–795. doi: 10.1080/03075079.2019.1652811

[ref37] Jung J. (2020). Master’s education in Hong Kong: access and programme diversity. High. Educ. Policy 33, 711–733. doi: 10.1057/s41307-020-00202-0

[ref38] Jung J. Lai M. Li X. (2023). Part-time master’s students’ attitudes towards study and work. Stud. Contin. Educ., 1–18. doi: 10.1080/0158037X.2023.2254244

[ref39] Jung J. Lee S. J. (2019). Exploring the factors of pursuing a master’s degree in South Korea. High. Educ. 78, 855–870. doi: 10.1007/s10734-019-00374-8

[ref40] Jung J. Li X. (2021). Exploring motivations of a master’s degree pursuit in Hong Kong. High. Educ. Q. 75, 321–332. doi: 10.1111/hequ.12276

[ref41] Jung J. Li X. Lai M. (2022). Concept of research among master’s students in Hong Kong. High. Educ. doi: 10.1007/s10734-022-00989-4

[ref42] Kong K. C. Kok L. H. Fernandez J. L. (2015). Factors that influence the decision to pursue studies or work after graduation: a study among final year undergraduates in Universiti Sains Malaysia. In: Conference Proceedings of Social Sciences Postgraduate International Seminar (SSPIS).

[ref43] Liu J. (2010). The changing body of students a study of the motives, expectations and preparedness of postgraduate marketing students. Mark. Intell. Plan. 28, 812–830. doi: 10.1108/02634501011086436

[ref44] Liu H. Lin Y. (2021). Factors influencing pharmaceutical engineering undergraduates to pursue graduate studies. Int. J. Eng. Educ. 37, 351–361.

[ref45] Liu D. Morgan W. J. (2016). Students’ decision-making about postgraduate education at G University in China: the main factors and the role of family and of teachers. Asia-Pacific Educ. Res. 25, 325–335. doi: 10.1007/s40299-015-0265-y

[ref46] Loes C. N. Pascarella E. T. (2015). The benefits of good teaching extend beyond course achievement. JoSoTL 15, 1–13. doi: 10.14434/josotl.v15i2.13167

[ref47] Ma E. Hsiao A. Gao J. Vada S. (2020). Inspiring good soldiers cross-culturally through the lens of the theory of planned behavior-which works best, norms or behavioral control? J. Hosp. Tour. Manag. 45, 99–112. doi: 10.1016/j.jhtm.2020.07.014

[ref48] Ma L. Lan Z. Tan R. (2020). Influencing factors of innovation and entrepreneurship education based on the theory of planned behavior. Int. J. Emerg. Technol. Learn. 15, 190–206. doi: 10.3991/ijet.v15i13.15345

[ref49] Mahagamage Y. Marasinghe K. (2023). The socio-economic effects of covid-19. Saúde Soc. 32, 1–12. doi: 10.1590/s0104-12902022200961en

[ref50] Makrygianni D. Koufaki M. Patrinos G. P. Vasileiou K. Z. (2023). Pharmacy students’ attitudes and intentions of pursuing postgraduate studies and training in pharmacogenomics and personalised medicine. Hum. Genomics 17:27. doi: 10.1186/s40246-023-00474-8, PMID: 36959668 PMC10035981

[ref51] Mullen A. L. Goyette K. A. Soares J. A. (2003). Who goes to graduate school? Social and academic correlates of educational continuation after college. Sociol. Educ. 76, 143–169. doi: 10.2307/3090274

[ref52] Naseer S. Khalid S. Parveen S. Abbass K. Song H. Achim M. V. (2023). COVID-19 outbreak: impact on global economy. Front. Public Health 10:1009393. doi: 10.3389/fpubh.2022.1009393, PMID: 36793360 PMC9923118

[ref53] Ni D. Guo F. Zhang H. Li M. Zhou Y. (2022). Improving older drivers’ behaviors using theory of planned behavior. Sustainability 14:4769. doi: 10.3390/su14084769

[ref54] Onyango G. (2023). The post-COVID-19 economic recovery, government performance and lived poverty conditions in Kenya. Public Organ. Rev., 1–19. doi: 10.1007/s11115-023-00732-2

[ref55] Pascarella E. T. (1985). Students’ affective development within the college environment. J. High. Educ. 56, 640–663. doi: 10.1080/00221546.1985.11778733

[ref56] Peng Y. Lv S. B. Low S. R. Bono S. A. (2023). The impact of employment stress on college students: psychological well-being during COVID-19 pandemic in China. Curr. Psychol. 68:101258, 1–12. doi: 10.1007/s12144-023-04785-w, PMID: 37359658 PMC10228455

[ref57] Posselt J. R. Grodsky E. (2017). Graduate education and social stratification. Annu. Rev. Sociol. 43, 353–378. doi: 10.1146/annurev-soc-081715-074324, PMID: 30662143 PMC6335048

[ref58] Rajeh M. T. (2022). Modeling the theory of planned behavior to predict adults’ intentions to improve oral health behaviors. BMC Public Health 22:1391. doi: 10.1186/s12889-022-13796-4, PMID: 35858885 PMC9297589

[ref59] Rembischevski P. Caldas E. D. (2020). Risk perception related to food. Food Sci. Tech. Brazil 40, 779–785. doi: 10.1590/fst.28219

[ref60] Rock L. D. Macdonald L. K. Compton S. M. MacDonald L. L. Kanji Z. (2023). Motivations for and outcomes of graduate education amongst dental hygienists: a pan-Canadian study. Int. J. Dent. Hyg. doi: 10.1111/idh.12726, PMID: 37635457

[ref61] Settersten R. A. Bernardi L. Härkönen J. Antonucci T. C. Dykstra P. A. Heckhausen J.. (2020). Understanding the effects of covid-19 through a life course lens. Adv. Life Course Res. 45:100360. doi: 10.1016/j.alcr.2020.100360, PMID: 36698274 PMC7375263

[ref62] Shah N. Soomro B. A. (2017). Investigating entrepreneurial intention among public sector university students of Pakistan. Educ. Train. 59, 841–855. doi: 10.1108/ET-11-2016-0168

[ref63] Shelley A. R. Veach M. C. Leroy B. Redlinger-Grosse K. (2020). A systematized review of experiences of individuals in arnett’s emerging adulthood stage who live with or are at-risk for genetic conditions. J. Genet. Couns. 29, 1059–1080. doi: 10.1002/jgc4.1238 32146730

[ref64] Shi H. (2023). The generation mechanism underlying the career decision-making difficulties faced by undergraduates in China during the COVID-19 pandemic: a qualitative study based on SCCT theory. Front. Psychol. 14:1154243. doi: 10.3389/fpsyg.2023.1154243 37377699 PMC10291063

[ref65] Siegrist M. Árvai J. (2020). Risk perception: reflections on 40 years of research. Risk Anal. 40, 2191–2206. doi: 10.1111/risa.13599, PMID: 32949022

[ref66] Slovic P. (1987). Perception of risk. Science 236, 280–285. doi: 10.1126/science.3563507 3563507

[ref67] Statista Research Department. (2022). Number of master’s degree recipients U.S. 1880–2032. Available at: https://www.statista.com/statistics/238236/masters-degree-recipients-in-the-us (Accessed November 7, 2023).

[ref68] Steinmayr R. Weidinger A. F. Schwinger M. Spinath B. (2019). The importance of students’ motivation for their academic achievement-replicating and extending previous findings. Front. Psychol. 10:1730. doi: 10.3389/fpsyg.2019.01730 31417459 PMC6685139

[ref69] Sun H. Zhang Q. Guo W. Lin K. (2022). Hikers’ pro-environmental behavior in national park: integrating theory of planned behavior and norm activation theory. Front. For. Glob. Change 5:1068960. doi: 10.3389/ffgc.2022.1068960

[ref70] Super D. E. (1980). A life-span, life-space approach to career development. J. Vocat. Behav. 16, 282–298. doi: 10.1016/0001-8791(80)90056-1

[ref71] Tang H. Rasool Z. Khan M. A. Khan A. I. Khan F. Ali H.. (2021). Factors affecting e-shopping behaviour: application of theory of planned behaviour. Behav. Neurol. 2021, 1–15. doi: 10.1155/2021/1664377, PMID: 34858540 PMC8632471

[ref72] Tett R. P. Burnett D. D. (2003). A personality trait-based interactionist model of job performance. J. Appl. Psychol. 88, 500–517. doi: 10.1037/0021-9010.88.3.500, PMID: 12814298

[ref73] Theodorou A. Hatzithomas L. Fotiadis T. Diamantidis A. Gasteratos A. (2023). The impact of the COVID-19 pandemic on online consumer behavior: applying the theory of planned behavior. Sustainability 15:2545. doi: 10.3390/su15032545

[ref74] Thomas S. D. Ali A. Alcover K. Augustin D. Wilson N. (2021). Social and professional impact of learning communities within the alliances for graduate education and the professoriate program at Michigan State University. Front. Psychol. 12:734414. doi: 10.3389/fpsyg.2021.734414, PMID: 34899474 PMC8654777

[ref75] To W. M. Lai L. S. L. Lung J. W. Y. Lai T. M. (2014). Intent to pursue further studies among Chinese students. Educ. Stud. 40, 292–309. doi: 10.1080/03055698.2014.889598

[ref76] Tsai K. Chou T. Kittikowit S. Hongsuchon T. Lin Y. Chen S. (2022). Extending theory of planned behavior to understand service-oriented organizational citizen behavior. Front. Psychol. 13:839688. doi: 10.3389/fpsyg.2022.839688, PMID: 35465519 PMC9024217

[ref77] Ulker-Demirel E. Ciftci G. (2020). A systematic literature review of the theory of planned behavior in tourism, leisure and hospitality management research. J. Hosp. Tour. Manag. 43, 209–219. doi: 10.1016/j.jhtm.2020.04.003

[ref78] Vassie L. Slovic P. Fischhoff B. Lichtenstein S. (2005). Facts and fears: understanding perceived risk. Policy Pract. Health Saf. 3, 65–102. doi: 10.1080/14774003.2005.11667668

[ref79] Wright E. Horta H. (2017). Higher education participation in “high-income” universal higher education systems: “survivalism” in the risk society. Asian Educ. Dev. Stud. 7, 184–204. doi: 10.1108/AEDS-07-2017-0061

[ref80] Xu Z. Li J. Yang Z. Shan J. (2021). Residents’ willingness to pay for the elimination of Ulva prolifera bloom: a case study in Qingdao, China. J. Environ. Plan. Manag. 64, 755–773. doi: 10.1080/09640568.2020.1784114

[ref81] Xue W. Wang L. Yang Z. Xiong Z. Li X. Xu Q.. (2023). Can clean heating effectively alleviate air pollution: an empirical study based on the plan for cleaner winter heating in northern China. Appl. Energ. 351:121923. doi: 10.1016/j.apenergy.2023.121923

[ref82] Yang C. Chan S. (2020). Massified master’s education in Taiwan: a credential game? High Educ. Policy 33, 619–635. doi: 10.1057/s41307-020-00213-x

[ref83] Yang S. Yang J. Yue L. Xu J. Liu X. Li W.. (2022). Impact of perception reduction of employment opportunities on employment pressure of college students under COVID-19 epidemic-joint moderating effects of employment policy support and job-searching self-efficacy. Front. Psychol. 13:986070. doi: 10.3389/fpsyg.2022.986070, PMID: 36337528 PMC9627308

[ref84] Yin L. Wu Y. J. (2023). Opportunities or threats? The role of entrepreneurial risk perception in shaping the entrepreneurial motivation. J. Risk Finan. Manag. 16:48. doi: 10.3390/jrfm16010048

[ref85] Yu T. Lavallee J. P. Di Giusto B. Chang I. Yu T. (2020). Risk perception and response toward climate change for higher education students in Taiwan. Environ. Sci. Pollut. R. 27, 24749–24759. doi: 10.1007/s11356-019-07450-7, PMID: 31900770

[ref86] Yuriev A. Dahmen M. Paillé P. Boiral O. Guillaumie L. (2020). Pro-environmental behaviors through the lens of the theory of planned behavior: a scoping review. Resour. Conserv. Recycl. 155:104660. doi: 10.1016/j.resconrec.2019.104660

[ref87] Zamfir A. Mocanu C. Davidescu A. A. (2021). What encourages longer educational careers in tertiary education? A three-level approach for the case of Romanian universities. Int. J. Environ. Res. Public Health 18:12864. doi: 10.3390/ijerph182312864, PMID: 34886590 PMC8657777

[ref88] Zhang S. Tang X. (2021). Cultural capital as class strength and gendered educational choices of Chinese female students in the United Kingdom. Front. Psychol. 11:584360. doi: 10.3389/fpsyg.2020.584360, PMID: 33536965 PMC7848284

[ref89] Zheng S. Wu G. Zhao J. Chen W. (2022). Impact of the COVID-19 epidemic anxiety on college students’ employment confidence and employment situation perception in China. Front. Psychol. 13:980634. doi: 10.3389/fpsyg.2022.980634, PMID: 36160584 PMC9501885

