# Supplementary material for: Factors that influence the intent to pursue a master’s degree: evidence from Shandong Province, China
Source: Front Psychol. 2024 Jan 12;15:1284277. doi: 10.3389/fpsyg.2024.1284277 (PMC10811022; doi:10.3389/fpsyg.2024.1284277)
Supplement: Supplementary file 1 [file Data_Sheet_1.docx]

Supplementary Information for

**Factors that influence the intent to pursue a master’s degree: Evidence from Shandong Province, China**

**Contents of this file**

Table S1 – S3

TABLE S1 Description of potential influencing factors.

| Variable | Sub-items | Description | Cronbach’s α |
| --- | --- | --- | --- |
| Attitude(AT) | A1 | I find postgraduate study beneficial for employment | 0.710 |
|  | A2 | I find postgraduate study beneficial for development and achievements in a certain field |  |
|  | A3 | I find postgraduate earnings are higher, and they are prone to promotion |  |
| Subjective norms(SN) | S1 | Parents encouraged me to take the postgraduate entrance examination | 0.719 |
|  | S2 | Teachers encouraged me to take the postgraduate entrance examination |  |
|  | S3 | Senior schoolmates encouraged me to take the postgraduate entrance examination |  |
| Perceived behavioral control(PBC) | P1 | I have enough knowledge to succeed in my postgraduate entrance examination | 0.744 |
|  | P2 | I have enough perseverance to succeed in my postgraduate entrance examination |  |
|  | P3 | I have enough comprehensive qualities to succeed in my postgraduate entrance examination |  |
| Risk perception(RP) | R1 | I am worried about failing the postgraduate entrance examination due to fierce competition | 0.826 |
|  | R2 | I am worried that preparation for the postgraduate entrance examination will harm my job hunting |  |
|  | R3 | I am worried that time spent in graduate study is not as rewarding as employment or entrepreneurship |  |
| Social factors(SO) | SO1 | I choose to take the postgraduate entrance examination because of growing employment difficulties due to the COVID-19 pandemic | 0.744 |
|  | SO2 | I choose to take the postgraduate entrance examination because of the degree inflation |  |
|  | SO3 | I choose to take the postgraduate entrance examination because of employers’ complex about famous universities |  |

TABLE S2 Respondents’ attributes.

| Attributes | Description |
| --- | --- |
| gender | 1=male, 0=female |
| graduation | The year of graduation ranges from 2023 to 2026 |
| honor | 1=received honors during undergraduate years,0= received no honors during undergraduate years |
| performance | academic performance ranges from 1 = low level to 5 =high level |
| internship | 1=got internship during undergraduate years,0= got no internship during undergraduate years |
| income | 1=family income less than 50,000 yuan,2=family income ranging from 50,000 to 150,000 yuan,3=family income ranging from 150,000 to 300,000 yuan,4=family income higher than 300,000 yuan |
| city | 1= home address in cities，0= home address in rural areas |

TABLE S3 Descriptive statistics.

| Variable | N | Mean | SD | Min | Max |
| --- | --- | --- | --- | --- | --- |
| Y | 417 | 4.237 | 0.802 | 1 | 5 |
| A1  A2 | 417  417 | 4.436  4.213 | 0.655  0.788 | 1  1 | 5  5 |
| A3 | 417 | 4.436 | 0.760 | 1 | 5 |
| S1 | 417 | 4.470 | 0.693 | 1 | 5 |
| S2 | 417 | 4.261 | 0.773 | 1 | 5 |
| S3 | 417 | 4.091 | 0.830 | 1 | 5 |
| P1 | 417 | 3.993 | 0.813 | 1 | 5 |
| P2 | 417 | 4.187 | 0.768 | 1 | 5 |
| P3 | 417 | 4.129 | 0.771 | 1 | 5 |
| R1 | 417 | 3.525 | 1.191 | 1 | 5 |
| R2 | 417 | 2.782 | 1.276 | 1 | 5 |
| R3 | 417 | 2.698 | 1.341 | 1 | 5 |
| SO1 | 417 | 3.609 | 1.058 | 1 | 5 |
| SO2 | 417 | 3.856 | 1.130 | 1 | 5 |
| SO3 | 417 | 3.688 | 1.132 | 1 | 5 |
| gender | 417 | 0.451 | 0.498 | 0 | 1 |
| graduation | 417 | 2024 | 0.950 | 2023 | 2026 |
| honor | 417 | 0.818 | 0.387 | 0 | 1 |
| performance | 417 | 4.065 | 0.755 | 2 | 5 |
| internship | 417 | 0.703 | 0.458 | 0 | 1 |
| income | 417 | 2.314 | 0.811 | 1 | 4 |
| city | 417 | 0.674 | 0.469 | 0 | 1 |
